# Reference-free SNP detection: dealing with the data deluge

**DOI:** 10.1186/1471-2164-15-S4-S10

**Published:** 2014-05-20

**Authors:** Richard M Leggett, Dan MacLean

**Affiliations:** 1 The Genome Analysis Centre, Norwich Research Park, NR4 7UH Norwich, UK; 2 The Sainsbury Laboratory, Norwich Research Park, NR4 7UH Norwich, UK

## Abstract

Reference-free SNP detection, that is identifying SNPs between samples directly from comparison of primary sequencing data with other primary sequencing data and not to a pre-assembled reference genome is an emergent and potentially disruptive technology that is beginning to open up new vistas in variant identification that reveals new applications in non-model organisms and metagenomics. The modern, effcient data structures these tools use enables researchers with a reference sequence to sample many more individuals with lower computing storage and processing overhead. In this article we will discuss the technologies and tools implementing reference-free SNP detection and the potential impact on studies of genetic variation in model and non-model organisms, metagenomics and personal genomics and medicine.

## SNP calling pipelines rely on a reference genome

It is no overstatement to say that the recent technological advances that have made it possible to sample whole genomes many times over has changed forever the way that geneticists and genomicists design and carry out their experiments. Many of these experiments require the detection of genetic variants as a preliminary, most often single nucleotide polymorphisms (SNPs). The basis of all these experiments is the same: one sequence must be compared with another. The predominant model for this is to have a single genome assembly chosen as the baseline against which all others will be compared. Often this reference genome will have been produced in a large-scale 'big-biology' project by a large consortium using long read Sanger-style sequencing or a hybrid approach that mixes long and short reads, but the defining characteristic will be that a great deal of time and expense has gone into preparing the reference. To avoid this expense later, the genome is not assembled for each further individual sample, instead the sequence reads will be aligned directly to the single assembled reference irrespective of any assembly errors or structure specific to the population or individual sequenced. The consensus from the sequence reads will then be taken as the new sample's genome and SNPs are called using sophisticated statistical methods based on sequence and alignment quality metrics.

## The De Bruijn graph as a data-structure for identifying variants

**Figure 1:**
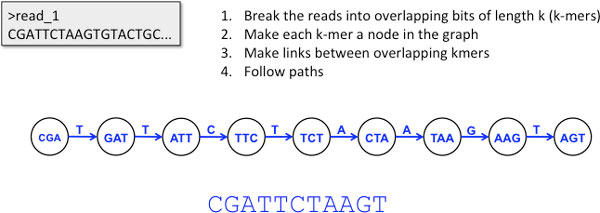
**De Bruin graphs constructed from overlapping k-mers**. De Bruijn graphs are networks of short overlapping sub-sequences of reads of length *k*. Typically, *k*-mers are set as the nodes in the graph and links are drawn between *k*-mers that have overlap of length *k - 1*, that is they overhang each other by just one nucleotide.

**Figure 2:**
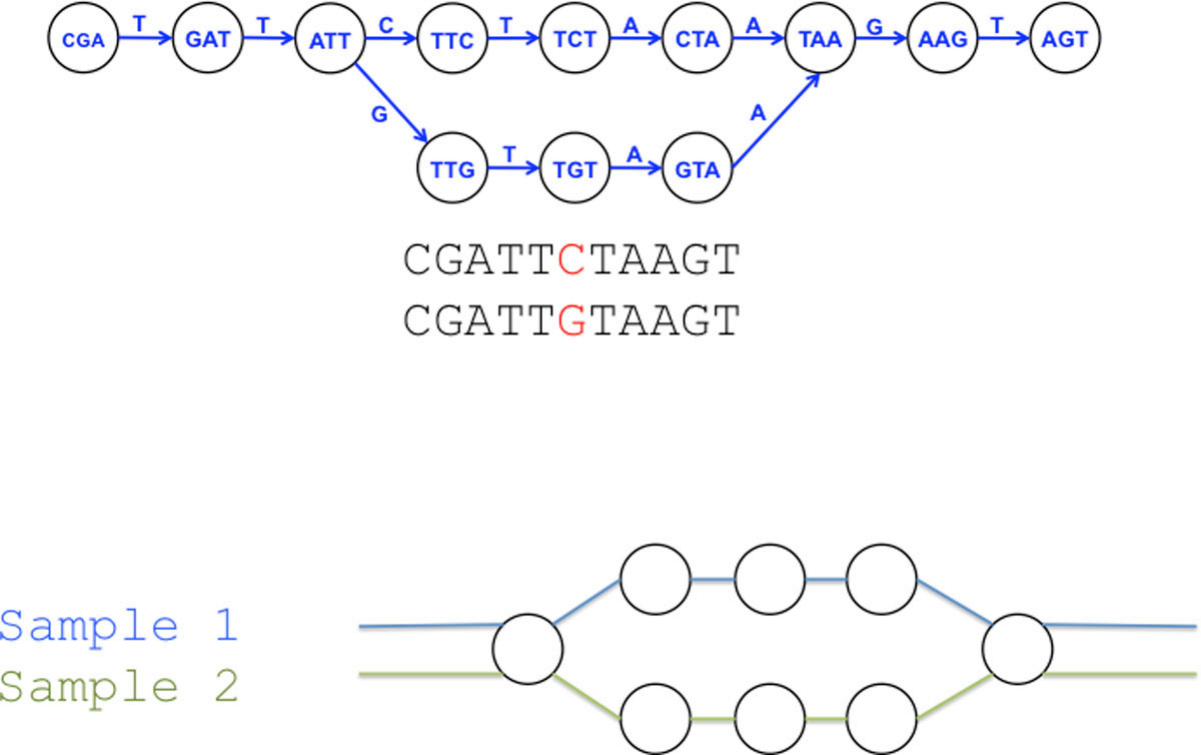
**Bubble structures formed in De Bruijn graphs by SNPs**. Bubble structures form as the result of a divergence in sequence by one nucleotide, initially at the end of a *k*-mer, that then moves backwards at each progressive node, allowing for a close of the two paths at the end. Colouring the edges in the graph according to sample provenance helps identify inter-sample SNPs.

Recently, a new model that is predominantly based on De Bruijn graphs, has emerged that removes the need for a reference genome and uses comparisons of the raw sequence directly. De Bruijn graphs are directed graphs of overlapping symbols that are well suited to representing ordered relationships between same length sequences, such as sub-sequences of sequence reads (see Figure 1). De Bruijn graphs have proven to be of great utility as the underlying data model over which almost all *de novo *assembly algorithms designed to use short read data have been implemented. Some examples of these tools are Velvet [1], ABySS [2], Euler [3], SOAPdenovo [4] and ALLPATHS [5]. In the majority of these assembly tools the De Bruijn graph is implemented as an internal data structure that represents a network in which the linked entities are *k*-mers from the sub-sequences of sequence reads and links are made between *k*-mers that overlap by *k*-1 with a single nucleotide overhang (in some implementations the *k*-mer is the link, rather than the entity). In a situation with no read errors and with *k *long enough to include the longest repeat in a single *k*-mer then it is theoretically possible to reconstruct the genome by following from *k*-mer to *k*-mer along the edges, passing through each *k*-mer only once. Since sequencing data do contain errors and genomic repeats can be very long (many times longer than frequently used read length, let alone the length of *k *used) then the read errors can cause dead ends in the path that terminate extension of the contig, repeats in the genome can cause cycles in the graph. True genetic variation will also cause these sub-structures. The different ways in which the De Bruijn graph based assembly programs get around these problems, essentially how they throw out variation-like structure in the graph, is what separates them and is the area on which assembler research is currently focussed. By focussing on these structures, rather than throwing them away, we can identify variation directly from the graph of *k*-mers. The basic distinct structure that indicates the presence of a SNP is described as a 'bubble' (see Figure 2). A bubble is caused by a single nucleotide difference at the end of two *k*-mers that causes a closing bifurcation within the graph such that each path around the bubble indicates a different allele. SNPs can be identified by searching the De Bruijn graph for these bubble structures. These bubbles may also have long leading or trailing paths of nucleotides, meaning SNPs can be embedded in long contigs.

## Limitations of short-read alignment based variant calling

With current tools the SNP content of whole genomes of many organisms can be compared very widely in just hours and the differences between them catalogued almost as a matter of course. As such we have vast new horizons of genetic variation to explore. The new vistas offered by advances in sequencing are not as broad as they might at first seem and there are significant disadvantages to the usual SNP- by-alignment approach.

A typical SNP and INDEL calling pipeline based on alignment to a reference is restricted by the reference. Generally, deletions and only small variations of that canonical sequence can be found whereas larger structural rearrangements, such as inversions or large insertions cannot be, without significant further analysis, such as reassembly of the genome or any non-mapping reads. The reads are too small to show these within themselves and any mate-pair orientation or read insert size information that points towards this will often be rejected since it does not fit in with the *a priori *quality parameters of the alignment pipeline. As a consequence sequence reads from such areas are rejected and the regions cannot be discovered. A reference-free *de novo *assembly approach can discover small changes like these as new or sample dependent sections in the graph. Reference-free methods are not the only approach and a number of techniques incorporating reference-based mapping have also been published that seek to identify more complex structural variations through additional algorithmic layers built on top of standard mapping tools such as BWA [6-8].

Multiple recent studies are now suggesting that an individual's genomes may be much more mosaic-like than previously thought [9] and that important variations may be a result of larger structural changes rather than simply substitutions in the sequence. A reference-free model allows us to find such variations as a series of portions of the graph apparent in only one or a few samples. Such approaches also offer greater power to identify rarer events, for example in comparisons of parental and child genomes and exomes, due to the algorithms giving less reliance to simple coverage based metrics.

Many scientists work with non-model organisms that lack an assembled reference genome, so when work that aims to identify variation between samples a typical workflow is to build a working reference genome. Completed or even relatively good drafts are time consuming and expensive to build from next generation sequencing technologies and as the genome sequence is only a means to an end in these cases then often primary contigs or first-pass scaffolds will be used. Ultimately, this can be of detriment to the final aim as poor quality and incomplete reference means that read alignment can be badly sub-optimal and generate many false positives or miss much of the variation. There are systematic errors that occur in alignment-based SNP detection that rely particularly on reference genome quality and the alignment parameters: three valid SNPs, equally-spaced in the read, can mean no SNPs found at all if the analysis specifies a two mismatch cutoff per read. A reference-free method that captures all information from the reads can potentially avoid the sequence problems of an error-prone early draft genome and reduce expense from the time and effort required to create one and therefore potentially maximise the power of variant calling.

Metagenomic data provide an even greater challenge to the task of SNP calling. Samples typically consist of a mixture of genomes at widely varying abundance levels. Even with extremely deep sequencing, the genomes of some species will be incomplete in the read set. The difficulty of estimating read depth, combined with the likelihood that many of the genomes will be from closely related species makes SNP calling based on depth extremely problematic. Thus, reference-free algorithms that take account of local graph topology or locally derived coverage statistics have the potential to produce results where a standard alignment based approach would fail.

Data storage and archiving is a major problem for alignment based methods of SNP calling. The major recording method for alignments is the SAM format, a record-per-alignment format whose file size increases with the number of reads that have been used in the alignment. Although clever compression and indexing to BAM format and others [10] alleviates some of the pain, the storage of alignment data for many samples consumes large amounts of expensive disk-space, takes a long time to move across networks for processing or sharing, and requires that storage continually expands as new samples are added. As these files can be many gigabytes in size for individual samples, it is not hard to see how a medical centre trying to deal with many hundreds of patients data in a clinical diagnostic setting would perhaps struggle to keep up with demands on that data. Nor is it hard to see how a population genomics study would need to rely on massive amounts of storage for its many data sets, thereby limiting the sample that could actually be taken and the science that could be done. Using a De Bruijn graph to hold sequence information can potentially be much more compact than the conventional per-read alignment in BAM files since each *k*-mer of the original sequence need only be represented once. Per sample counts of occurence of that *k*-mer are recorded and the structure of the graph encoded. New *k*-mers not seen in previous samples increase the size of the files more substantially as they, their counts and the changes to the graph structure must be recorded, but the overall growth is many times less than that taken up by per-alignment storage methods. A significant disadvantage of the De Bruijn graph approaches developed so far is that individual read information is lost in the preparation of the graph. Scientists still like to be able to check each read manually when calling SNPs, often to convince themselves of the quality of the calls and rule out obvious errors in SNP calling in a very *ad hoc *manner, reads cannot yet be reconstituted from De Bruijn graphs as they can from BAM alignments so a dual storage is needed. Although the cost of sequencing has stopped declining at the rate it has done over the last few years, the low cost of the actual sequence is still a major attraction to laboratories interested in these sorts of experiments. The cost of analysis and storage of data is much more often overlooked and constitutes a substantial investment too. While it is true that for alignment-based SNP detection it is arguable that we need only store BAM files for as long as it takes to calculate a result and that final output can be stored more efficiently than the reads, such an approach precludes the possibility of truly novel discovery that the De Bruijn graph approach makes possible and is also not amenable to easy re-analysis.

Although being able to call SNPs and other variants without a reference is very useful, when a reference is available it can add much to analyses using these systems as it can provide a very useable guide set of *k*-mers that SNP finding without losing any of the advantages of the reference-free model.

## Strategies for identifying variants from the structure of a De Bruijn graph

### Depth-first

Iqbal *et al*. produced the first methods for detecting variants and distinguishing populations or individuals by finding alleles with the use of a differential 'colouring' technique on the graph [11]. A key component of their strategy is to add a nominal colour to the *k*-mers that come from each sample so that uniquely 'coloured' segments in the graph then represent regions unique to one sample, naturally this approach is well suited to describing insertions or deletions as well as SNPs. Variant identification from a De Bruijn graph relies on finding the structures that correspond to different classes of variant. Since the SNPs manifest as bubble structures within the graph, one way of finding these structures is to use a graph traversal algorithm, such as the clean bubble caller employed in Iqbal *et al*., and the more computationally intensive depth-first search method in Leggett *et al*. [12]. These algorithms proceed by marking the nodes in the graph that have at least two edges departing from them as starting points and following nodes sequentially (graph walking) until a node with at least two edges entering it is reached. Two such paths from the same origin to the same destination, with different colours delineate a potential bubble and therefore SNP between samples (Figure 2). A complication occurs when the paths split again before they can resolve, resulting in an large increase in search space and therefore compute time. In the case of Iqbal *et al*., path complexity can be restricted with the use of a reference, while the solution for Leggett *et al*. is to limit the number of branching paths that are followed with a maximum depth parameter and thereby allow the algorithm to complete. A significant source of false-positives for this method comes from the fact that dual out-edges can be created by genome structure that is not just a SNP- any pair of *k*-mers that differ by a single end nucleotide causes the key bifurcation thus repeats, inversions and other low-complexity genome sequence can be problematic to differentiate from the desired SNPs.

This approach is not just restricted to identifying SNPs. Iqbal *et al *also showed the power of the approach for finding INDELs and larger scale variants between samples, though power of the algorithm is increased for complex INDELs by using a reference.

### Topological

Another graph structure, or topological method that has appeared recently has taken a very strict approach to the definition of the bubble structures and therefore represents a potentially very effective SNP detection tool. In the 2*k *+ 2 approach, bubbles are identified by decomposing the De Bruijn graph to an undirected graph and then identifying bubbles as Eulerian and Hamiltonian cycles, i.e paths in bubble sized subgraphs (2*k*) that visit each node and edge just once on their way back to the starting node. This method's very precise description of a bubble means that it has the potential to be extremely specific and generate highly accurate lists of SNPs from graph structure alone (Younsi *et al*., unpublished) (2*k *+ 2), however the computational time required is great and sensitivity must be compromised, the entire graph cannot be searched and random start sites within the graph must be selected repeatedly to prevent ample coverage, furthermore as graph complexity increases the time to complete increases badly too, rendering these approaches likely unsuitable for metagenomics applications or situations were completeness of SNP detection, rather than simpler marker selection is needed.

### Microassembly

Peterlongo *et al*. [13] adopt a microassembly approach to SNP detection. They begin by producing a tree of *k*-mers for an input read set and then build bubble microassemblies (or 'mouth' structures in their terminology) based on the *k*-mer set. The algorithm consists of first picking a seed *k*-mer by choosing the *k*-mer with the lowest count that occurs more in one set than another. A low count *k*-mer is chosen to avoid repeat structures, while it his hoped that a differing set count may be indicative of a SNP. Having chosen a seed, the algorithm assumes that it lies on one path through a SNP and then looks for an opposite *k*-mer, one substitution different, which would lie on another path through the bubble. If this can be found in the *k*-mer tree, then a recursive algorithm builds paths left and right of each *k*-mer until they join up or no *k*-mer can be found in the tree. Once a bubble has been identified, it is checked for read coherence by checking for coverage from at least two reads. The approach has a significant memory saving and speed advantage over techniques that build a complete De Bruijn graph, as the initial tree structure does not include connectivity information. However, the method does not handle heterozygous SNPs and is limited to pairwise comparisons for data sets containing more than two samples. An improved version of the technique is currently being prepared for publication that addresses some of these limitations ([14] and R. Uricaru, *pers. comm*.).

### Maximum likelihood methods

The Stacks tool developed by Catchen *et al*. [15] is designed for restriction enzyme based sequencing protocols such as RAD-Seq (Restriction site Associated DNA), a technique designed to sample from the same reduced and representational fraction of the genomes of multiple individuals of a population [16,17]. The method begins by generating 'stacks' of reads at particular loci, which form because of the nature of the restriction enzymes used. These stacks are then merged with nearby stacks using either a *de novo *or a reference based approach. SNPs are called from stacks using a single-nucleotide, diploid maximum liklihood genotyping algorithm designed for RAD-Seq data by Hohenlohe *et al*. [18]. Related work by Dou *et al*. take this ML method further, publishing what is described as an improved maximum liklihood algorithm (iML) [19]. They note that in a Stacks experiment, short reads from repetetive regions cluster together and are difficult to distinguish, potentially resulting in false SNP calling. They observe that the distribution of composite clusters should show a repeating pattern corresponding to copy number variations of the repeating elements. This is modelled with a Poisson distribution and the model combined with the Hohenlohe *et al*. ML algorithm to exclude repetetive loci from SNP calling.

### Contig based graphs

Approaches specifically targetted at metagenomic data have been rare, due to the complexity of such datasets. However Nijkamp *et al*. have recently published a tool called MARYGOLD which aims to identify complex bubbles in and between metagenomic samples [20]. Their approach is based on contig graphs rather than De Bruijn graphs. In such graphs, the nodes represent contigs and the edges represent reads that span connected contigs. In metagenomic datasets, multiple edges indicate sequence divergence of the same or related species and, as with De Bruijn graphs, these edges typically reconverge forming bubble structures. The MARYGOLD approach is to identify these regions and collapse them to form contigs representing multiple alleles. The contigs are output in a graph structure that represents the multiple alleles and output in Circos format is also supported.

## Implementations

There are already a number of tools already available for reference-free SNP calling (see Table 1). The most significant tool yet implemented for reference free analysis is Cortex - in which Iqbal *et al *implemented fast and highly memory efficient data structures and algorithms for general variant detection, and is the first implementation that makes use of explicitly coloured De Bruijn graphs and incorporates a *de novo *assembly algorithm. Given a specific genome of interest, a variant-size and a *k*-mer size, the power to detect variants was modelled, interestingly the authors noted that without a reference, it was difficult to distinguish read errors and repeats from genuine SNPs. An extension module to Cortex named Bubbleparse handles this problem explicitly by incorporating extra information on genetic background and applying a maximum likelihood heuristic to rank candidate bubbles and thereby increase accuracy of SNP calling so that false-postive calls can be reduced by five-fold relative to the base algorithm, moving from 50 - 60 % false positive unranked to5 - 10 % after ranking. Bubbleparse can also select and prioritise SNPs that satisfy prior information about genetic background, meaning that it is particularly suited to the identification of SNPs *de novo *from specific genetic crosses.

**Table 1:** *de novo *reference-free analysis software and availability.

Cortex	http://cortexassembler.sourceforge.net/
Bubbleparse	https://github.com/richardmleggett/bubbleparse
Bubbleparse accessories	https://github.com/danmaclean/Bubble-Parse
2*k *+2	http://sourceforge.net/projects/twokplustwo/
NIKS	http://sourceforge.net/projects/niks/
discoSnp	http://colibread.inria.fr/discosnp/
Stacks	http://creskolab.uoregon.edu/stacks/
MaryGold	http://sourceforge.net/projects/metavar/

Pipelines have also been developed for the identification of polymorphisms *de novo *and for tracking of genetic loci from populations sequenced using the RAD tag approach, a reduced representation sequencing process, Stacks [15] is a comprehensive toolkit that uses a non De Bruijn graph method and instead uses sample categorisation (equivalent to colours in a De Bruijn graph) to group reads from the tags that are decomposed into a *k*-mer list, or stack. A similarity graph between stacks is created to reduce their number and to merge stacks to give the potential SNP loci. A maximum likelihood consensus is used to infer the SNPs and genetic cross data is incorporated to construct haplotypes and create comprehensive, dense genetic maps.

The more recent NIKS, needle in a *k *stack, tool [21] is a related but much more directed procedure that does not aim to call a wide range of SNPs or produce a map. Rather it aims to narrow down the SNPs that correspond to mutations induced after a mutant screen and sequencing from simple genetic back crosses, especially those performed for purifying a mutation after EMS mutagenesis of the model plant *Arabidopsis thaliana*. Non-candidate SNPs are discarded by the method.

The 'mouth' work of Peterlongo *et al *resulted in the tool kisSnp, which evolved into kisSnp2 and is now part of discoSnp [14,22]. discoSnp combines the SNP predictions from kisSnp2 with a second module which computes mean coverage and average quality per read set of the reads generating the SNP.

## Impact of reference-free SNP calling on the biomedical sciences

The impact of reference-free SNP calling promises to be significant. Genetic studies in non-model organisms will be facilitated greatly - with these methods it is now possible to start assaying genetic variability across many samples without the time and expense of building a reference genome. This alone brings the technology to smaller groups with fewer resources but also allows new questions to be asked across wider phylogenetic groupings than is possible. Although the reference free methods are just as sensitive to nucleotide distance between samples and large genetic distances cannot be assayed any more effectively than with alignment based methods, virtually any organism can be compared against close relatives with just the sequencing and much lower computational overhead. Since many of the reference-free tools are based on the same De Bruijn graph structure as *de novo *short read assemblers the SNP calls generated can be embedded in a long species-specific contig, an attribute that promises to have great use in the field of plant breeding. Domesticated crop species typically have very large, highly repetitive genomes that have been refractory to attempts to generate completed genome sequences. A key aim in plant breeding is to identify the genetic variation that contributes to agronomically important traits so that it can be selected effectively and introduced into commercially important crop lines. Closely related wild relatives of crop species, a great example is wheat and related wild grasses, are reservoirs of genes that can confer resistance to pathogens. By using reference-free SNP approaches plant breeders can access a much wider pool of resistance related genes since the alleles that associate with resistance in some individuals or populations can be assayed much more quickly. The ability to generate contigs with trait linked SNPs embedded means standard tools like BLAST can be used to identify the candidate function of the gene from which that contig arose, even when no specific genetic resources are available.

Application of reference-free SNP detection methods and data structures could help to resolve the data storage and access issues associated with alignment-based methods meaning that true genome wide SNP and variant searches can be more easily commoditised for the medical and clinical markets and therefore be a crucial element in the deployment of personal genomics based medicine. Plummeting sequence prices are starting to make individual personal genome sequencing a diagnostic reality. The small data overhead incurred for each new sample sequenced means much more information can be stored for similar cost which brings new diagnostic and research possibilities much closer. Being able to work with sequence regions not present in the available reference genomes and the SNPs in those regions means that new associations between disease and genetic variations can be collected, and the reference free framework is fit to make this research possible directly from the clinic. Variant detection absolutely *de novo *like this means that the complex mutations that make up the genomic pathologies of cancers, for example, can be uncovered much more easily for each patient and case and the changes cross-referenced with the patients own individual reference. Furthermore the recent realisations that our genomes vary even within us, perhaps from tissue to tissue is much more tractable in the reference-free framework.

We are learning that individual organisms genomes can be incredibly diverse and capturing that diversity will become vital in future research. The reference-free SNP detection methods are among the first steps in a new approach that will help relieve us from the limiting prior information in the reference genome.

## Competing interests

The authors declare that they have no competing interests.

## Authors' contributions

RML and DM equally contributed to the text.

## Declarations

The publication costs for this article were funded by a grant from the BBSRC

This article has been published as part of *BMC Genomics *Volume 15 Supplement 4, 2014: SNP-SIG 2013: Identification and annotation of genetic variants in the context of structure, function, and disease. The full contents of the supplement are available online at http://www.biomedcentral.com/bmcgenomics/supplements/15/S4
